# Recruiting and exploring vulnerabilities among young people at risk, or in the early stages of serious mental illness (borderline personality disorder and first episode psychosis)

**DOI:** 10.3389/fpsyt.2022.943509

**Published:** 2022-08-04

**Authors:** Ruchika Gajwani, Naomi Wilson, Rebecca Nelson, Andrew Gumley, Michael Smith, Helen Minnis

**Affiliations:** ^1^Institute of Health & Wellbeing, University of Glasgow, Glasgow, United Kingdom; ^2^NHS Greater Glasgow and Clyde, Glasgow, United Kingdom

**Keywords:** severe mental illness (SMI), borderline personality pathology, psychosis, emotional dysregulation, adverse child experiences, neurodevelopmental disorders

## Abstract

**Introduction:**

Many gaps exist in our understanding of the developmental pathways to severe mental illness (SMI), including borderline personality disorder (BPD) and psychosis. However, those who have experienced adverse childhood experiences (ACEs) are at an increased risk and there is evidence to suggest that one of the earliest markers is emotional dysregulation. An area which has received relatively less research attention is the role neurodevelopmental disorders (NDDs) play. The aim of this feasibility study was therefore to explore the clinical profiles of young people early in the course of SMI, including their profiles of ACEs, emotional regulation difficulties, borderline personality traits and NDDs.

**Methods:**

A cross-sectional study of young people (aged 15–25) at risk of SMI, currently being seen within NHS mental health services, was conducted. This included those with early symptoms of psychosis and/or BPD as assessed by diagnostic interview. Eligible participants self-completed a battery of sociodemographic, clinical, and psychological measures in the company of a researcher. This included assessments of: symptoms of NDDs; borderline pathology traits; ACEs; and difficulties in emotional regulation. Statistical analyses included Mann–Whitney U tests and multiple regression.

**Results:**

Of the 118 potentially eligible participants who were referred, 48 were ultimately included in the study. Young people early in the course of SMI reported a high prevalence of ACEs and deficits in emotional regulation. In total, 79% met criteria for attention deficit hyperactivity disorder (ADHD) and/or autism spectrum disorder (ASD). Emotional dysregulation was found to significantly mediate the association between both ACEs and the frequency of NDDs and borderline personality traits, however given the small sample size these results are preliminary in nature.

**Conclusion:**

Young people early in the course of SMI are at an increased risk of experiencing multiple childhood adversities and our results indicate a high prevalence of NDDs amongst them. Emotional dysregulation emerged as a potentially significant early marker of future clinical severity. We suggest that the clinical implications of our findings include routine screening for NDDs and ACEs and an increased recognition of the significance of emotional dysregulation. However, larger scale longitudinal studies are needed to investigate these preliminary findings further.

## Introduction

Severe mental illness (SMI) commonly refers to any distinct psychiatric diagnosis in adulthood which is both severe and substantially functionally impairing (1). Examples include schizophrenia, bipolar affective disorder and borderline personality disorder (BPD). Once established, such conditions are often viewed as intractable (2) and have severe individual, familial and societal consequences (3). It is now well established that the onset of SMI peaks during the transition from childhood to adulthood, with 75% of longstanding and persistent SMI’s starting between the ages of 10 and 24 (4). Importantly, it is also clear that the symptoms of SMI are more malleable to treatment in this age group (5). Identifying early features of potential SMI and understanding the common trajectories of adolescents and young adults at risk is therefore a research imperative.

Many gaps exist in our understanding of the developmental pathways to SMI (6). Specifically, we are currently unable to accurately predict which “at risk” young people will go on to develop which specific disorder (6). However, what is becoming clear is that the earliest clinical presentation of a range of potential SMI’s can often be a complex collection of undifferentiated symptoms and general psychopathology (7, 8). For example, a recent epidemiological study and clinical staging model identified that young people at high risk of a range of mood and psychotic spectrum disorders have broadly similar symptomatology and are not easily distinguishable (9). These manifestations can be disabling, yet, due to the current focus on adult taxonomy and diagnoses, youth with early symptoms are often missed by services and left untreated (10). Indeed, even when young people receive “a diagnosis,” that diagnosis frequently changes (11, 12), emphasizing the importance of studying the evolution of SMI comprehensively, and with attention to the unique needs of the young person.

Despite this, accumulating evidence does now suggest that one of the most frequent early markers of potential SMI is emotional dysregulation (13, 14). For example, large epidemiological studies have identified high levels of emotional dysregulation in those at high risk, and in the early stages of both psychosis (15) and BPD (16). Emotional dysregulation is also a core characteristic of multiple established SMI diagnoses, including BPD (17), bipolar affective disorder, eating disorders, and psychotic spectrum disorders (18, 19). Specific links between psychotic symptoms and BPD through emotional dysregulation have also been identified (20), with empirical evidence for the co-occurrence of the two syndromes (21). Specifically, psychotic symptoms in individuals with a BPD diagnosis have been shown to be exacerbated by situational or interpersonal stress responses (15, 22).

Emotional dysregulation is also a valuable focus for investigation because of the light it may shed on developmental pathways from environmental risk to psychiatric illness. Emotional regulation is a developmental task and a developmental experience, highly influenced by the opportunity to develop secure attachments in early life, through experiences of responsive and attuned caregiving (23). Early risk markers of insecure attachment, such as childhood trauma and neglect, are known to significantly increase the risk of developing emotional dysregulation and subsequent poor health outcomes in later life (24), although the causal pathways are far from simple. It has therefore been suggested that emotional dysregulation may represent a key mediating pathway between early exposure to childhood adversity and the subsequent development of SMI in adult life (25). Investigating the emotional regulation profiles of “at risk” youth is therefore of critical importance.

An area which until recently has received less research attention is the role neurodevelopmental disorders (NDDs), such as attention deficit hyperactivity disorder (ADHD) and autism spectrum disorder (ASD), play in the development of SMI. The association between NDD in childhood and SMI in adulthood is increasingly recognized. Recent findings have shown that ASD symptoms are common in adults with a diagnosis of schizophrenia (26) and that both ASD and ADHD are highly prevalent in those with a diagnosis of BPD (27, 28). However, some studies also suggest that NDDs are underdiagnosed generally (29) and are frequently missed and undertreated in children with a history of childhood abuse or neglect (30). This is despite recent evidence to indicate that having a NDD increases the odds that a child will be exposed to maltreatment (31) and that, vice versa, children who have been maltreated are more likely to present with coexisting NDDs (32). Further exploration of the prevalence of NDDs among clinical samples early in the course of SMI is therefore essential to understanding the scale of diagnostic overshadowing which may be occurring amongst those with a history of maltreatment. In addition, one of several hypothesized reasons NDDs are often missed in this population is the significant symptomatic overlap which exists between childhood trauma-related disorders and NDDs (33). Specifically, emotional dysregulation is a core feature of both (34). There would therefore also be great value in improving our understanding of the role emotional regulation plays in the relationship between childhood trauma, NDDs and SMI. Such insights are crucial to both advancing our understanding of the pathogenesis of SMI and to identifying potential targets for early intervention.

### Aims

The primary aim of this pilot study was to determine the overall feasibility of recruiting and retaining a sample of young people (age 15–25) early in the course of SMI from a range of primary and secondary mental health services. For the purposes of this study, this included those with early onset psychosis (clinical high-risk of psychosis and first episode psychosis) and/or those early in the course of BPD (including those with sub-threshold symptoms).

Our secondary aims were: (1) to explore the sociodemographic and clinical profile of this group, including their profiles of adverse childhood experiences (ACEs), emotional regulation difficulties and borderline personality traits; (2) to investigate the prevalence of common NDDs, specifically ASD and ADHD; and (3) to determine whether emotional dysregulation mediates the relationship between either frequency of ACEs or frequency of NDDs and the severity of borderline personality traits among this group.

Finally, in this study, we examine three specific hypotheses:

1. Young people early in the course of SMI with comorbid NDD will present with a profile of greater rates of ACEs and emotional dysregulation.
2. Emotional dysregulation will mediate any association between the frequency of ACEs and the severity of borderline personality traits in young people early in the course of SMI.
3. Emotional dysregulation will also mediate any association between the frequency of NDDs and the severity of borderline personality traits in young people early in the course of SMI.

## Materials and methods

### Study design and setting

A cross-sectional study of young people at risk of SMI, currently being seen within NHS mental health services in the United Kingdom, was conducted.

The protocol for the study was developed in 2016 and data collection took place over a 24-month period between 2016 and 2018. Ethical approval for the study was gained from an NHS ethics committee, United Kingdom (Ethics Ref.: 16/WS/0133). All study participants gave written informed consent and could withdraw from the study at any point without treatment being affected.

### Participants

Young people aged between 15 and 25 years of age, who met one of the below four inclusion criteria were eligible to participate. For participants under the age of 16, a parent or legal guardian was required to provide written consent on their behalf.

1. Were identified as having subsyndromal symptoms of BPD, by meeting between 2 and 4 of 9 criteria for the BPD section of the Structured Clinical Interview for DSM-5: Personality Disorders (SCID-II) [Thompson et al. (35)].

OR

2. Had an established diagnosis of BPD, using the DSM-5 criteria of 5 out of 9 criteria on the SCID II (BPD module).

OR

3. Were identified as at Ultra-High Risk (UHR) of psychosis according to the comprehensive assessment of the at-risk mental state using the CAARMS) [Yung et al. (36)].

OR

4. Had previously been diagnosed with a first episode of psychosis (FEP). This was assessed with the CAARMS.

Non-English speakers were excluded.

All assessments relating to eligibility were completed through a clinician led or supervised diagnostic interview. Findings are presented for the complete sample (*N* = 48) of young people at risk of SMI and for the subgroups (BPD/subsyndromal BPD and FEP/UHR) in Supplementary Tables 1, 2 provided.

### Sampling and recruitment

The study was presented to numerous national health service (NHS) mental health teams across the Greater Glasgow and Clyde Health Board. Potentially eligible participants were then referred by a mental health professional to the study from a range of those services, including: Community Mental Health Teams (CMHTs), Primary Care Mental Health Teams (PCMHTs), Clinical Psychology Services, Community Adolescent Mental Health Services (CAMHS), and Personality Disorder Teams (AMHS).

### Procedure and setting

Following their referral to the study potential participants were contacted by a member of the research team to arrange an appointment. At the first appointment, informed written consent was sought, a purposely designed sociodemographic and clinical questionnaire was self-completed by participants and assessments relating to eligibility (CAARMS and SCID-II) were then completed through a clinician led/supervised diagnostic interview.

At a second visit, all remaining measures were completed by participants themselves, in the company of a member of the research team. This was conducted in a number of settings, including the clinic of the referring mental health team, or, if required, in their home, if the patient’s clinician concluded it was safe to do.

### Measures

#### Primary measures/clinical assessments

1. To establish early psychosis criteria (UHR or FEP), the CAARMS interview was administered. Participants were recruited into the early psychosis group if they met criteria (a) at-risk mental states (ARMS) criteria for attenuated symptoms of psychosis, (b) family history of psychosis and a decline in functioning using the global assessment of functioning tool (GAF), and (c) ARMS BLIPs-group (brief limited intermittent *psychotic* symptoms) or (d) FEP criteria on the DSM-5.
2. To establish early BPD criteria (subthreshold or full-threshold), all potentially eligible participants were interviewed using the Structured Clinical Interview for DSM-5 Axis II Personality Disorders (SCID-II) BPD module. Participants were recruited to the early BPD group if they met sub-threshold (2 or up to 4 out of 9 domains) or threshold (5 and above out of 9 domains) criteria on the SCID-II DSM-5.

#### Secondary measures

1. *Sociodemographic and clinical items:* A purposely designed sociodemographic and clinical questionnaire was self-completed by participants. This included three sociodemographic items and three clinical items. Sociodemographic items were participants age, gender, and the presence or absence of parental psychopathology. Clinical items were the presence or absence of: any episodes of deliberate self-harm in the previous 2 weeks; any previous psychiatric admissions; or any previous suicide attempts.
2. *Adverse childhood experiences:* The ACEs Questionnaire was used to screen for a history of childhood maltreatment. This 10-item self-report measure assesses 10 types of childhood trauma measured in the ACE Study. Five are personal (including physical or emotional abuse or neglect and sexual abuse) and five are related to household dysfunction (including exposure to domestic violence, parental mental illness, substance misuse, incarceration, or divorce) (37).
3. *Emotional regulation difficulties:* Emotional regulation was assessed according to the Difficulties in Emotional Regulation Scale (DERS). This 36-item, self-report measure assesses six facets of emotion regulation, including: acceptance of emotional responses; impulse control; emotional awareness; and access to emotional regulation strategies. Items are rated on a scale of 1 (“almost never”) to 5 (“almost always”). There are no clinical cut off scores, however a higher score indicates greater difficulty in emotional regulation.
4. *Borderline personality traits:* Borderline personality traits was assessed using the Borderline Personality Questionnaire (BPQ). This self-report screening tool has been specifically developed and validated for the assessment of BPD in young people according to DSM-5 criteria. It consists of 80 true/false statements and has repeatedly demonstrated good sensitivity and specificity in predicting BPD (38).
5. *Neurodevelopmental disorders:* Two self-report symptom scales were used to screen for ADHD and ASD respectively.
  a. *ADHD Self-Report Scale (ASRS):* This self-report screening scale includes 18 questions about frequency of recent DSM-5 Criterion A symptoms of adult ADHD. It has been shown to be a reliable and valid scale for evaluating ADHD in adults and shows high internal consistency and high concurrent validity with the rater-administered ADHD Report Scale.
  b. *Autism Symptom SElf-ReporT for adolescents and adults (ASSERT):* This seven item self-report tool is used to screen for ASD in adolescents and young adults.

### Statistical analysis

Statistical analysis was completed using SPSS V24. Type I error rate was set to α = 0.05. Data was analyzed for normal distribution using Kolmogorov–Smirnov test. Non-parametric tests (the Mann–Whitney U test) were subsequently used to analyze differences in borderline personality traits, emotional regulation difficulties, and ACEs between those who did and did not screen positively for a NDD. To test the mediating relationship between NDDs and ACEs (i.e., predictor variables) and borderline personality traits (i.e., outcome variable) with emotional regulation as the mediating variable, multiple regression analysis was conducted.

## Results

**FIGURE 1:**
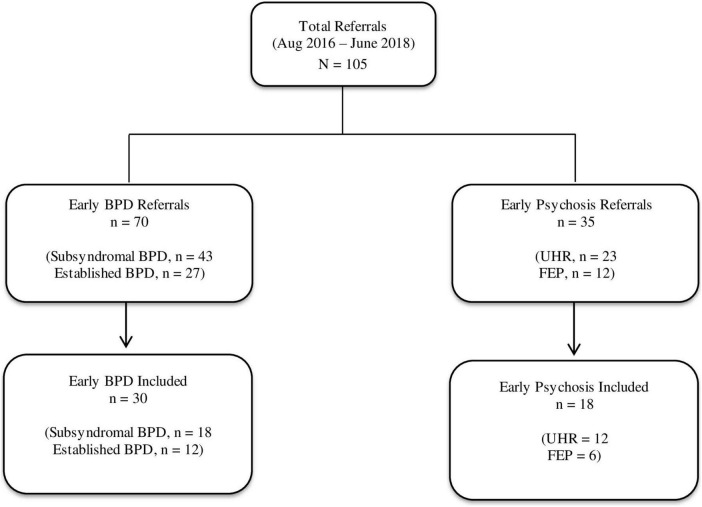
Participant flow diagram.

Of the 118 potentially eligible participants who were referred for screening, 105 were deemed eligible to participate. This included 70 participants with early BPD (including 43 participants identified as having subsyndromal symptoms and 27 identified as having established BPD) and 35 participants with early psychosis (including 12 participants who were identified as UHR and 6 who were identified as having FEP). 48 participants subsequently consented and completed self-report outcomes measures at a second appointment and were therefore ultimately included in the study (Figure 1).

### Sociodemographic features and psychiatric history of the sample

Table 1 presents the sociodemographic profile and psychiatric history of the complete sample (*N* = 48) and the two sub-groups in Supplementary Table 1. The majority of participants were female and between the ages of 15 and 20. Parental psychopathology was reported by 64% of participants and close to half (48%) had had a previous psychiatric admission. Nearly three quarters of participants (74%) reported having attempted suicide within their lifetime and 38% reported having deliberately self-harmed in the past 2 weeks.

**TABLE 1:** Sociodemographic and clinical profile of the sample.

Variable	Number of participants (percentage %)
Total sample	48 (100%)
**Gender**	
Female	38 (79%)
Male	10 (21%)
**Age**	
15–20 years	28 (58%)
21–25 years	9 (19%)
26–35 years	11 (23%)
**Parental psychopathology**	
Yes	30 (64%)
No	17 (36%)
(1, 2%)*	
**Previous psychiatric admission**	
Yes	23 (48%)
No	25 (52%)
**Previous self-harm (last 2 weeks)**	
Yes	18 (38%)
No	30 (62%)
**Previous suicide attempt (lifetime)**	
Yes	28 (74%)
No	10 (26%)
(10, 21%)*	
**Adverse childhood experiences (ACEs)**	
0	9 (20%)
1	8 (17%)
2	3 (4%)
3	5 (11%)
>4	22 (48%)
(2, 4%)*	
**ACE abuse by category**	
Emotional	22 (48%)
Physical	18 (39%)
Sexual	13 (28%)
**Neglect by category**	
Emotional	8 (17%)
Physical	13 (28%)
**Household dysfunction by category**	
Parental divorce/separation	22 (48%)
Domestic violence	12 (26%)
Mental illness in household	28 (61%)
House-member in prison	9 (20%)
Household substance misuse	19 (41%)

*Number of participants with missing data for this item, percentage of cases this represents.

Overall, 80% (*n* = 37) of the sample reported experiencing one or more ACEs, with 48% (*n* = 22) reporting four or more ACEs. The most common form of childhood adversity was parental mental illness (61%), followed by emotional abuse (48%), and parental divorce/separation (48%). Approximately 65% of the sample indicated some impairment in functioning (≤50 on the GAF).

### Emotional regulation difficulties and borderline personality traits

Possible DERS scores range from 36 to 180. Although no clinical cut-off score exists, published values span from 63.68 amongst healthy controls; to 93.42 in bipolar disorder; and 108.24 in a group of participants with depression (13). In the current study, the overall sample mean was reported at 126.0 (SD = 25.4) suggesting significant deficits in emotional regulation abilities amongst participants as a whole. Sub-sample findings are reported in Supplementary Table 2, however, the early BPD group mean was reported at 129 and the early psychosis group mean was reported at 120.88.

The total sample mean for BPQ was 47.15 (SD = 15.37). In total, 19 (41%) participants also met the cut off score for BPD on the BPQ (i.e., a score of 56 or more), which is reported to have moderate sensitivity, high specificity, and overall diagnostic accuracy.

### Prevalence of neurodevelopmental disorders

Thirteen participants (28%) in this study had a previous diagnosis of either ASD or ADHD, described here as NDD diagnosis. All 13 participants with a diagnosis also screened positive on the NDD self-report assessments, indicating no false negatives. Of the 48 participants in the sample as a whole, 34 (71%) screened positive for ADHD, 25 (53%) screened positive for ASD, and 20 (42%) screened positive for both ADHD and ASD. Two participants had incomplete data for these measures.

Table 2 shows descriptive data of borderline personality traits, emotion regulation, and frequencies of ACEs according to NDD screening outcome. That is those who screened positively for ADHD and/or ASD (*n* = 38) and those who screened negatively for ADHD and ASD (*n* = 8).

**TABLE 2:** BBQ, DERS, and ACEs scores by NDD screening result.

Variables	NDD screening result		Mann–Whitney U test	
		
	NDD +ve (*n* = 38)	NDD −ve (*n* = 8)		
		
	Mdn (IQR)	Mdn (IQR)	U	Sig.
BPQ	55.0 (23.0)	35.0 (29.0)	219.5	0.005
DERS	135.0 (27.8)	108.0 (32.3)	246.0	0.04
ACEs	3.00 (5.00)	3.50 (5.00)	160.0	0.83

BPQ, Borderline Personality Questionnaire; DERS, Difficulties in Emotional Regulation Scale; ACEs, adverse childhood experiences; NDD, neurodevelopmental disorders.

Significant differences in borderline personality traits and emotional regulation were identified between these groups. Specifically, those who screened positively for either ADHD or ASD had significantly higher DERS scores and scores for borderline personality traits than those who screened negatively for either (Table 2). No significant differences in ACEs were identified between the NDD groups.

### Emotional dysregulation as a mediator of the relationship between adverse childhood experiences or neurodevelopmental disorders and borderline personality traits

Finally, mediation analyses were conducted to determine the role of emotional dysregulation (DERS) (*mediator variable*) in mediating (a) the relationship between frequency of ACEs (*predictor variable*) and borderline personality traits (BPQ) (*outcome variable*) or (b) frequency of NDD’s (*predictor variable*) and borderline personality traits (*outcome variable*). Three steps of regression analysis were carried out to test these mediating relationships as recommended by Baron and Kenny (39). Unstandardized estimates are reported as recommended by Preacher and Hayes (40). Given the small sample size, these analyses are preliminary in nature and should be interpreted with caution.

**FIGURE 2:**
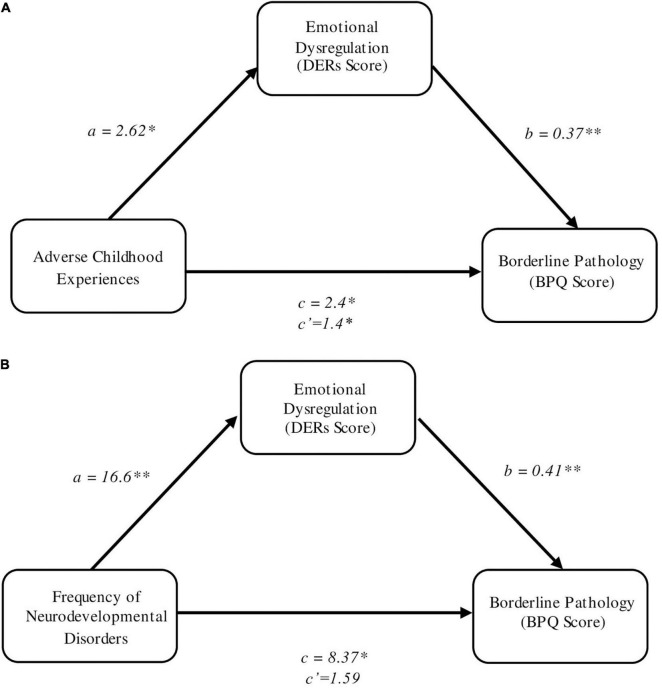
**(A)** Emotional dysregulation as a mediator of the relationship between adverse childhood experiences and borderline personality pathology. **(B)** Emotional dysregulation as a mediator of the relationship between frequency of NDDs and borderline personality pathology. Unstandardized coefficients are reported. *p ≤ 0.05; **p<0.01.

In step 1 of our first analysis, regression identified a significant relationship between frequency of ACE’s and borderline personality traits (*c* path) (*B* = 2.4, SE = 0.69, *t* = 3.49, *p* = 0.001). In step 2, the “*a*” path was also found to be significant, with the frequency of ACEs being significantly associated with emotional dysregulation (*B* = 2.62, SE = 1.25, *t* = 2.1, *p* = 0.04). In step 3, borderline personality traits was regressed onto emotional dysregulation and the frequency of ACE’s. This identified, the “*b*” path was also significant, with emotional dysregulation being significantly associated with borderline personality traits (*B* = 0.37, SE = 0.06, *t* = 5.8, *p* < 0.001). After accounting for emotional dysregulation, the direct effect of the frequency of ACEs on borderline personality traits (c′ path) remained significant (*B* = 1.4, SE = 0.54, *t* = 2.6, *p* = 0.012), supporting partial atemporal mediation. A Sobel test was conducted which subsequently identified this partial mediation was significant (*z* = 1.98, SE = 0.49, *p* = 0.047) (Figure 2A).

In the second mediation analyses, we explored whether emotional dysregulation mediated the association between frequency of NDDs (i.e., no NDDs; one NDD, either ADHD or ASD; or two NDDs, both ADHD and ASD) and severity of borderline personality traits. Regression analysis again identified a significant relationship between the frequency of NDD’s and borderline personality traits (*c* path) (*B* = 8.37, SE = 2.85, *t* = 2.94, *p* = 0.005). The “*a*” path was subsequently also found to be significant, with the frequency of NDD’s being significantly associated with emotional dysregulation (*B* = 16.6, SE = 4.5, *t* = 3.68, *p* < 0.001). Finally, borderline personality traits was regressed onto both emotional dysregulation and the frequency of NDDs. This identified, the “*b*” path was also significant, with emotional dysregulation being significantly associated with borderline personality traits (*B* = 0.41, SE = 0.07, *t* = 5.6, *p* < 0.001). However, after accounting for emotional dysregulation, the direct effect of the frequency of NDDs on borderline personality traits (c′ path) was no longer significant (*B* = 1.59, SE = 2.5, *t* = 0.63, *p* = 0.53), suggesting that the relationship between the NDD screening result and borderline personality traits is fully mediated by emotional dysregulation in this sample. A Sobel test subsequently identified this mediating role as significant (*z* = 3.12, SE = 2.18, *p* = 0.0018) (Figure 2B).

## Discussion

This study aimed to examine the feasibility of recruiting and investigating the sociodemographic and clinical profiles of a group of young people early in the course of SMI. Given the limited data which exists on this population, the purpose of this was to gain a better understanding of their general presentation, and thus contribute to the growing knowledge base surrounding early markers of potential SMI. Our results are therefore of importance to furthering both the identification and treatment of young people most at risk and have produced several notable findings.

In the first instance, the sociodemographic profiles and psychiatric histories of the participating young people identified high levels of functional impairment and distress in the form of high prevalence rates of lifetime suicide attempts, recent deliberate self-harm and previous psychiatric admissions. These findings are in keeping with a growing body of research which demonstrates the considerable functional impairment and psychological distress amongst youth with attenuated symptoms of SMI, often in the absence of any “diagnosable” mental illness (41). For example, other epidemiological studies of young people with attenuated symptoms of SMI, but no “discrete disorder” have similarly identified high rates of unemployment, self-injurious behavior, and previous suicide attempts (9). As such, early intervention for this at-risk group may not only improve long-term outcomes, but could also reduce morbidity, mortality and improve present functioning in the short-term.

Eighty percent of the sample reported experiences of at least one ACE, and approximately half the sample reported four or more ACE’s. The most common form of adversity experienced was parental mental illness, with over sixty percent of the sample reporting parental psychopathology. The role of parental psychopathology is complex. It influences parent-child reciprocal roles, might reflect a genetic predisposition or vulnerability in the form of genetic and environmental interaction and acts as an independent risk factor for offspring psychopathology (42). However, overall this underlines the well-established role of ACEs in the development of SMI and highlights the need for routine screening of ACEs amongst those presenting to mental health services.

Secondly, our findings support the assertion that NDD’s are under-diagnosed in this population. Thirteen of the 48 participants (28%) had a previous diagnosis of NDD (ASD or ADHD). In contrast, 71% screened positively for ADHD; 53% screened positively for ASD; and 42% screened positively for both. Unfortunately, the study was unable to investigate the proportion of individuals that may not meet full diagnostic criteria upon further investigation. However, the outcome measures utilized have previously shown good diagnostic validity and reliability. As such, it is reasonable to assume that diagnostic overshadowing had occurred for some of those who screened positively. We would therefore recommend comprehensive screening assessments for neurodevelopmental difficulties are conducted routinely among young people at risk of SMI. Young people in our study who screened positively for an NDD, also had significantly higher levels of emotional dysregulation and borderline personality traits than those who screened negatively for any NDD. This should be interpreted with caution due to the small sample size. However, it is in keeping with growing evidence on the high levels of symptomatic overlap between NDDs and other SMIs, namely BPD. This can result in significant diagnostic challenges for clinicians. Further research is needed to determine the degree of comorbidity as opposed to symptomatic overlap which exists in this population.

Finally, our findings shed some light on the potential pathways between both childhood trauma and NDDs, and BPD as one form of SMI. Specifically, our results indicate that in this sample, the relationship between childhood adversity and borderline personality traits is partially mediated by emotional dysregulation, while the association between the frequency of NDD’s and borderline personality is *fully* mediated by emotional dysregulation. Moreover, mean scores for emotional dysregulation in the sample as a whole were higher than norms published for both clinical and non-clinical samples (43, 44). This is important as it supports existing evidence to suggest that emotional dysregulation is a potential early marker of SMI in both children with NDDs and children who have been exposed to maltreatment. It also indicates that this is likely to be a common pathway to SMI among young people at risk and as such, highlights it as a possible target for early intervention. Research suggests that emotional instability continues to be viewed as normative among adolescents by many clinicians (45) and it is therefore a symptom which, in isolation, does not often result in clinical intervention (46). However, while a degree of emotional dysregulation is consistent with normal development, studies have demonstrated that the extent and severity of emotional instability among young people early in the course of SMI makes it non-normative (47). Our data supports these findings and highlights the importance of recognizing and treating extensive or severe emotional dysregulation early, in order to prevent long-term morbidity and mortality.

### Strengths and limitations

The primary aim of this study was to examine is its overall feasibility. With a consent rate of approximately 40% of the participants referred, the data indicates several challenges to recruitment. However, once consented to the study, there were no withdrawals and the completion rate of the assessments was high, with incomplete data on only two participants (4%). In addition, the highest proportion of participants were recruited from specialist children’s services, supporting the growing evidence base for early detection and intervention for borderline psychopathology and psychosis in adolescence.

Nonetheless, it must also be noted that this pilot study had several limitations. It was not possible to rule out false positives in relation high rates of self-endorsed NDD’s. In addition, the presence of ACEs and NDDs overlap substantially, across generations and individually. Given the overlap, it is unknown whether ACEs add anything to the prediction of BPD and psychosis in young adults in the presence of NDDs or vice versa. However, we were unable to test the additive effects of the two risk factors on symptom outcomes in this pilot study due to its cross-sectional nature and small sample size. Moreover, the role of NDDs in other attenuated syndromes or other forms of “at risk” presentations was not explored. In particular young people with early mood symptoms, at potential risk of recurrent major depression or bipolar affective disorder, were not accounted for. Further studies would therefore benefit from a larger sample size, broader inclusion criteria and multi-informant diagnosis to confirm the prevalence of NDDs.

Finally, as highlighted, the data used in the present study was cross-sectional in nature, which precludes inferences that can be made about the direction of the associations between NDDs, emotion dysregulation, and borderline personality traits. In addition, our sample size was small and mediation analyses are therefore likely to be without sufficient power. As such, these are of a preliminary nature only and should be interpreted with caution. Previous research suggests that emotion dysregulation is both a cause and a consequence of BPD (48). It is therefore possible that pre-existing BPD may also mediate the relationship between emotional dysregulation and NDDs. Future research should use a prospective longitudinal design to elucidate the temporal relationship between NDDs, emotion dysregulation, and BPD or psychosis.

### Clinical implications

Taking into account the strengths and limitations of this feasibility study, our findings provide initial evidence that standardized clinical assessment of psychiatric comorbidities and NDDs should be conducted amongst all youth at risk of SMI. This would provide basis for a good clinical formulation and wrap around care planning, in addition to any planned intervention. Secondly, clinicians need to be aware of the increased risk for abuse or neglect in children with multiple NDDs, and vice versa.

Conducting comprehensive clinical assessments could potentially impact treatment – for example, an individual with undiagnosed NDDs and a trauma history may present as high risk to self with intrusive thoughts and may not receive appropriate treatment for NDDs if misdiagnosed. Finally, we that suggest that extensive or severe emotional dysregulation should be more readily recognized as a potential marker of future SMI and as a symptom which warrants intervention.

## Conclusion

Identifying early features of potential SMI and understanding the common trajectories of adolescents and young adults at risk is a research imperative. Our results indicate this is a group marked by significant social and clinical complexity, with an increased risk of experiencing multiple childhood adversities and a high prevalence of NDDs. Emotional dysregulation emerged as a potentially significant early marker of future clinical severity. We suggest that the clinical implications of our findings include routine screening for NDDs and experiences of maltreatment amongst children presenting to clinical services and recognition of the significance of emotional dysregulation as a potential early marker of risk of SMI. However, larger scale longitudinal studies are needed to investigate these preliminary findings further.

## Data availability statement

The raw data supporting the conclusions of this article will be made available by the authors, without undue reservation.

## Ethics statement

This study was reviewed and approved by an NHS Ethics Committee, United Kingdom (Ethics Ref.: 16/WS/0133). Written informed consent to participate in this study was provided by the participants or their legal guardian/next of kin.

## Author contributions

RG: study concept and design, interpretation, writing, supervision, and funding acquisition. NW: data analysis, interpretation, writing, and editing. RN: provision of study materials or patients, collection, and assembly of data. AG: study design, provision of study material or patients, supervision, and review. MS: study design, funding acquisition, and review. HM: study concept and design, provision of study material or patients, supervision, and editing. All authors contributed to the article and approved the submitted version.
