# Supplementary material for: Recruiting and exploring vulnerabilities among young people at risk, or in the early stages of serious mental illness (borderline personality disorder and first episode psychosis)
Source: Front Psychiatry. 2022 Aug 4;13:943509. doi: 10.3389/fpsyt.2022.943509 (PMC9386049; doi:10.3389/fpsyt.2022.943509)
Supplement: Supplementary file 1 [file Table_1.docx]

Supplementary Table 1. Clinical and Demographic Profile of Whole Sample and Subgroups

| **Variable** | **Complete sample**  **n = 48** | **Early BPD**  **[BPD(SS) + BPD]**  **n = 30** | **Early Psychosis**  **(UHR + FEP)**  **n = 18** |
| --- | --- | --- | --- |
| **Age (Mean, SD)** | 20.02 (5.6) | 19.73 (6.3) | 20.53 (4.3) |
| **Gender (n, %)**  Female  Male | 38 (79%)  10 (21%) | 24 (80%)  6 (20%) | 14 (78%)  4 (22%) |
| **Parental Psychopathology (n, %)**  Yes  No  (1, 2%)* | 30 (64%)  17 (36%) | 19 (65%)  10 (35%) | 11 (61%)  7 (39%) |
| **Psychiatric admission (n, %)**  Yes  No | 23 (48%)  25 (52%) | 16 (53%)  14 (47%) | 7 (39%)  11 (61%) |
| **Self-harm (last 2 weeks) (n, %)**  Yes  No | 18 (38%)  30 (62%) | 16 (53%)  14 (47%) | 2 (11%)  16 (89%) |
| **Suicidal attempts (lifetime) (n, %)**  Yes  No  (10, 21%)* | 28 (74%)  10 (26%) | 19 (90%)  2 (10%) | 9 (53%)  8 (47%) |
| **ACEs (n, %)**  0  1  2  3  >4  (2, 4%)* | 9 (20%)  8 (17%)  2 (4%)  5 (11%)  22 (48%) | 4 (14%)  5 (17%)  1 (4%)  5 (17%)  14 (48%) | 5 (29%)  3 (18%)  1 (6%)  0 (0%)  8 (47%) |
| **ACE Abuse by category (n, %)**  Emotional  Physical  Sexual  **Neglect by category (n, %)**  Emotional  Physical  **Household dysfunction (n, %)**  Parental divorce/sep.  Domestic violence  MI in household  House-member prisoned  Household sub. misuse | 22 (48%)  18 (39%)  13 (28%)  8 (17%)  13 (28%)  22 (48%)  12 (26%)  28 (61%)  9 (20%)  19 (41%) | 17 (59%)  13 (45%)  12 (41%)  5 (17%)  9 (31%)  13 (45%)  8 (28%)  20 (69%)  5 (17%)  12 (41%) | 5 (29%)  5 (29%)  1 (6%)  3 (18%)  4 (24%)  9 (53%)  4 (24%)  8 (47%)  4 (24%)  7 (41%) |

Note:

BPD(SS) = Borderline Personality Disorder (SubSyndromal); BPD = Borderline Personality Disorder; UHR = Ultra High Risk of Psychosis; FEP = First Episode Psychosis; n = number of participants; % = percentage of participants; SD = Standard Deviation.

*(number of participants with missing data for this item, percentage of cases this represents)
