# Supplementary material for: Recruiting and exploring vulnerabilities among young people at risk, or in the early stages of serious mental illness (borderline personality disorder and first episode psychosis)
Source: Front Psychiatry. 2022 Aug 4;13:943509. doi: 10.3389/fpsyt.2022.943509 (PMC9386049; doi:10.3389/fpsyt.2022.943509)
Supplement: Supplementary file 2 [file Table_2.docx]

Supplementary Table 2. Borderline Personality Traits and Emotional Regulation Difficulties in Whole Sample and Subgroups

| **Variable Mean (SD)**  (n missing data,  Percentage of cases) | **Early BPD**  **[BPD(SS) + BPD]**  **n = 30** | **Early Psychosis**  **(UHR + FEP)**  **n = 18** | **Range** |
| --- | --- | --- | --- |
| **BPQ (cut off >56)**  Yes  No  (2, 4%)* | 14 (48%)  15 (52%) | 5 (29%)  12 (71%) |  |
| **BPQ** | 52.10 (12.04) | 38.71 (17.05) | 0-80 |
| **DERS**  (2, 4%)* | 129 (21.20) | 120.88 (31.43) | 36-180 |

Note:

BPD (SS), Borderline Personality Disorder (SubSyndromal); BPD, Borderline Personality Disorder; UHR, Ultra High Risk of Psychosis; FEP, First Episode Psychosis; BPQ, Borderline Personality Questionnaire; DERS, Difficulties in Emotional Regulation Scale.

**(number of participants with missing data for this item, percentage of cases this represents)
